# Diagnostic value of shear wave elastography combined with super microvascular imaging for BI-RADS 3-5 nodules

**DOI:** 10.3389/fonc.2023.1192630

**Published:** 2023-09-05

**Authors:** Xueqing Wang, Yi He, Liangyu Wang

**Affiliations:** Department of Ultrasound, Shantou Central Hospital, Shantou, Guangdong, China

**Keywords:** breast cancer, breast nodules, shear wave elastography, super microvascular imaging, microvascular morphology classification

## Abstract

**Background:**

To investigate the diagnostic value of shear wave elastography (SWE) and super microvascular imaging (SMI) integrated with the traditional ultrasound breast imaging reporting and data system (BI-RADS) classification in differentiating between benign and malignant breast nodules.

**Methods:**

For analysis, 88 patients with 110 breast nodules assessed as BI-RADS 3-5 by conventional ultrasound were selected. SWE and SMI evaluations were conducted separately, and all nodules were verified as benign or malignant ones by pathology. Receiver operating characteristic (ROC) curves were plotted after obtaining quantitative parameters of different shear waves of nodules, including maximum (Emax), mean (Emean), minimum (Emin) Young’s modulus, modulus standard deviation (SD), and modulus ratio (Eratio). The best cut-off value, specificity, sensitivity, accuracy, positive predictive value (PPV), and negative predictive value (NPV) for diagnosing malignant nodules employing Emax were obtained, and the diagnostic value of combining Emax and BI-RADS classification was compared. SMI graded nodule based on the Alder blood flow grading standard, whereas the BI-RADS classification was based on microvascular morphology. We assessed the diagnostic value of SMI for breast nodules and investigated the diagnostic efficacy of SWE combined with SMI in differentiating benign and malignant breast nodules with BI-RADS classification 3–5.

**Results:**

The adjusted the BI-RADS classification using SMI and SWE technologies promoted the sensitivity, specificity, and accuracy of discriminating benign and malignant breast nodules (P < 0.05). The combination of traditional ultrasound BI-RADS classification with SWE and SMI technologies offered high sensitivity, specificity, accuracy, PPV, and NPV for identifying benign and malignant breast lesions. Moreover, combining SWE and SMI technologies with the adjusted BI-RADS classificationhad the best diagnostic efficacy for distinguishing benign and malignant breast nodules with BI-RADS 3–5.

**Conclusion:**

The combination of SWE and SMI with the adjusted BI-RADS classification is a promising diagnostic method for differentiating benign and malignant breast nodules.

## 1 Introduction

In clinical practice, imaging approaches including ultrasound, mammography, CT, and MRI are often employed for breast cancer screening. Mammography has been the preferred choice for early screening of breast cancer in the past (1–3). However, the sensitivity of mammography for detecting dense breast nodules is low (4, 5). Traditional ultrasound evaluation has become the preferred screening approach for the early detection of breast nodules because of its simplicity, safety, noninvasiveness, radiation-free, and real-time dynamic imaging benefits. However, breast nodules have various appearances on grayscale ultrasound images, and benign and malignant features overlap; therefore, the application of new ultrasound technologies is urgently required to enhance diagnostic efficacy (6–9).

SWE is a non-invasive, real-time, dynamic ultrasound imaging technology that evaluates tissue hardness by measuring the velocity of shear wave propagation in the target tissue. The principle is that the propagation speed of the mechanical wave is proportional to the hardness of the propagating medium, and the ultrasonic probe forms a continuous ultrasonic shear wave source in the target region, and then the propagation speed of the shear wave is accurately measured to calculate the hardness of the propagating medium. The relationship between shear wave velocity and microstructure hardness is positive, and the higher the velocity, the greater the microstructure hardness.Since malignant nodules are harder than benign ones, studies have demonstrated that the average shear wave velocity of malignant lesions is substantially higher than that of benign lesions (10–12). Breast tumors comprise various tissue components, primarily consisting of tumor cells and surrounding stromal components. In the growth process of malignant breast tumors, tumor cells constantly proliferate, infiltrate, necrose, and repair, resulting in collagen synthesis and fibrous tissue proliferation and reactive proliferation of surrounding connective tissue (13, 14). The higher the density of tumor cells, the more edema in the surrounding tissue and the higher the hardness of the tumor. This forms the pathological histological basis for employing elastography to differentiate between benign and malignant breast nodules (15, 16).

SMI technology is a real-time non-invasive microvascular imaging technology, which can detect low-velocity microvessels with high resolution, high frame rate and minimum motion artifacts. It adopts a unique adaptive algorithm to eliminate clutter and motion artifacts generated by source tissue motion through a multi-dimensional wall filter, thus minimizing the loss of low-speed blood flow information. Compared to traditional blood flow imaging (such as color Doppler and power Doppler), which can only show vessels with higher flow velocity and tube diameter > 0.2mm, SMI can visualize low-velocity tiny vessels with tube diameter > 0.1mm without injecting contrast agent. It represents a technological innovation in vascular imaging and is mainly used for the assessment of tumor vessels (17). Investigations have reported that it can substantially enhance the diagnostic effectiveness of benign and malignant breast nodules (18). The growth, invasion, and metastasis of breast cancer are closely associated with the formation of new microvessels. Therefore, the emergence and use of ultramicro vascular imaging technology offer a good complement to traditional ultrasound blood flow detection methods. It can not only measure low-speed microvessels with diameters greater than 0.1 mm but can also effectively separate low-flow signals from tissue motion artifacts, even retaining the finest low-flow components.

SWE and super microvascular imaging (SMI) technologies were combined in this research to reclassify breast imaging reporting and data system (BI-RADS) categories based on the hardness information and blood flow signal characteristics of breast nodules and to further examine the diagnostic value of the two new technologies for distinguishing between benign and malignant breast nodules classified as BI-RADS 3-5.

## 2 Materials and methods

### 2.1 Study population

Patients who visited the Central Hospital of Shantou fbecause of breast nodules were chosen as the study population. Inclusion criteria were as follows: (a) BI-RADS classification, SWE, and SMI diagnostic examinations were conducted with no contraindications; (b) complete imaging data were available. Exclusion criteria were as follows: (1) patients with a history of breast surgery, breast cancer recurrence, or concomitant malignant tumors; (2) lesions larger than 4 cm in maximum diameter or with internal liquefaction; (3) patients receiving neoadjuvant chemotherapy; (4) patients with a history of breast implantation; (5) lactating or pregnant women; (6) patients with mental or cognitive disorders. All lesions had undergone biopsy or surgical pathological diagnosis. 88 patients with 110 lesions were included, with ages ranging from 15 to 87 years and a mean age of (46.39 ± 14.81) years. The maximum diameter of breast lesions ranged from 4 to 40 mm, with a mean of (19.34 ± 7.96) mm.

### 2.2 Instruments and methods

#### 2.2.1 Instruments

A Japanese TOSHIBA Aplioi900 ultrasound diagnostic system equipped with SWE and SMI imaging technology with a high-frequency linear array probe of 10–14 MHZ was used. The “BREAST” mode of the instrument system settings was chosen. An ultrasound physician with over 10 years of experience and proficiency in SWE and SMI technologies guided the operator, and three operations were repeated to obtain an average value and reduce human error.

#### 2.2.2 Methods

First, a two-dimensional (2D) gray-scale breast ultrasound evaluation was conducted. The ultrasound probe was gently placed on the breast and radially scanned from the nipple. This process was repeated twice. The depth, gain, and focus were adjusted according to the lesion condition after detecting the breast lesion to obtain the best image quality. Sonographic features of the breast lesion were recorded in detail, and any abnormal lymph nodes in the axilla were also screened.

#### 2.2.3 Classification criteria and imaging observation indicators

Breast BI-RADS classification: The comprehensive analysis was conducted in accordance with the classification criteria of the 2013 version of the Breast Imaging Reporting and Data System (BI-RADS). Class 3: High probability of benign or low grade malignancy, the probability of malignancy is 0%-2%, short-term follow-up of 3 to 6 months; Class 4: Suspected malignancy, the possibility of malignancy is 3%-94%, biopsy is recommended; Class 5: Highly suggestive of malignancy, malignant probability ≥ 95%, recommended biopsy and active treatment. BI-RADS class 4 lesions were further classified into 4a, 4b, and 4c subcategories (18–20), as follows: 4a - low suspicion for malignancy, with a 3-10% likelihood of malignancy; 4b - intermediate suspicion for malignancy, with a 10-50% likelihood of malignancy; 4c - high suspicion for malignancy, with a 51-94% likelihood of malignancy. Malignant signs, such as microcalcifications, irregular shape, spiculated margins, were categorized, round shape, microlobulated/indistinct/angular margins, duct extension, complex echogenicity and posterior acoustic shadowing, non-parallel growth, were categorized (19, 20).

SMI: The number, course, and distribution of microvessels inside and around the lesion are observed using CDFI and mSMI technology after determining the location of the breast lesion using routine ultrasound. The size of the sampling box was modified, including regulating the blood flow velocity measurement range within 1 cm around the nodule and its surrounding breast tissue as much as possible, which is approximately 1.0–2.0 cm/s. To classify the microvascular morphology of breast nodules in SMI mode, Adler’s blood flow grading standard (21) was employed (22): (1) avascular type: no visible blood flow signal was detected within the nodule; (2) linear type: a single linear or slightly curved blood flow signal was detected within the nodule without crossing; (3) branching type: blood flow signals with uniform vessel diameter and branching were detected within the nodule, similar to branching; (4) root type: the blood vessel course within the nodule is irregular and disordered, and less than two large twisted blood vessels can be detected around it; (5) crab claw type: two or more radiating, thick and twisted blood vessels, or tiny, thorn-like blood vessels can be detected around the nodule. The microvascular morphology distribution was classified based on the above types after obtaining the mSMI blood flow image of the benign and malignant breast nodules (23). Among them, nodules with microvascular morphology types of avascular, linear, and branching were judged as benign nodules, and those with residual root and crab claw types were judged as malignant nodules.

SWE examination: SWE mode was initiated by the same physician using the same ultrasound diagnostic equipment after verifying the location of the lesion using traditional ultrasound (24–26). The measuring range was set to 0–180 kPa. The ROI was drawn to include the entire nodule using a grayscale ultrasound display of the lesion boundary. The SWE elasticity average value (Emean) of the lesion was measured. Then, the ROI range was set to 2 mm × 2 mm and placed in the elasticity mode map to obtain different SWE parameters including Emax, Emin, Eratio, SD, and Emean. Each data was measured three times and the average value was taken.

### 2.3 Statistics

For analysis, SPSS19.0 software was employed. Count data were presented as mean ± standard deviation (x ± s), whereas t-tests and chi-square tests were employed for continuous and categorical data, respectively. The sensitivity, specificity, positive predictive value (PPV), negative predictive value (NPV), and diagnostic accuracy for the diagnosis of breast nodules employing the BI-RADS classification, SWE, and SMI technology alone, and the two technologies combined with the BI-RADS classification diagnostic criteria, were separately computed. To compare the diagnostic indicators among different methods, chi-square tests were employed. Furthermore, receiver operating characteristic (ROC) curves were separately constructed for SWE, SMI, BI-RADS classification, and their combinations, and the area under the curve (AUC) was computed. The optimal cutoff value was determined as the elastic value with the maximum Youden index. A statistically significant difference was considered when P < 0.05.

## 3 Results

### 3.1 Pathological results

In this research, pathological findings were obtained from 110 solid nodules in 88 patients, which were either obtained through biopsy or surgical excision (**Table 1**). Of these, 60 nodules (54.54%) were malignant and 50 nodules (45.45%) were benign (**Table 1**).

**Table 1:** Pathological results of 110 breast nodules.

Pathological Results	Number (n)	Percentage (%)
Benign	50	
Fibroadenoma	37	33.63
Breast adenosis	5	4.54
Benign phyllodes tumor	3	2.72
Intraductal papilloma	3	2.72
Breast inflammation	2	1.81
Malignant	60	
Invasive ductal carcinoma	48	43.63
Invasive lobular carcinoma	3	2.72
Ductal carcinoma in situ	4	3.63
Mucinous carcinoma	3	2.72
Medullary carcinoma	1	0.90
Invasive small cell carcinoma	1	0.90
Total	110	

### 3.2 Conventional ultrasound BI-RADS classification results

The traditional ultrasound evaluation reported that among the 110 breast nodules, 26 (23.63%) nodules were categorized as BI-RADS 3 and were all benign; 14 (12.72%) nodules were categorized as BI-RADS 4a, with 2 (1.81%) nodules being malignant and 12 (10.90%) nodules being benign; 27 (24.54%) nodules were categorized as BI-RADS 4b, with 17 (15.45%) nodules being malignant and 10 (9.09%) nodules being benign; 32 (29.09%) nodules were categorized as BI-RADS 4c, with 30 (27.27%) nodules being malignant and 2 (1.81%)nodules being benign; 11 (10%)nodules were categorized as BI-RADS 5 and were all malignant. BI-RADS 3-4a lesions were considered benign, while BI-RADS 4b-5 lesions were considered malignant. There were two cases of infiltrating ductal carcinoma among the misdiagnosed malignant nodules. There were six cases of fibroadenoma, four cases of mammary gland hyperplasia, one case of chronic mastitis, and one case of lymphocytic mastitis among the misdiagnosed benign lesions.

### 3.3 Examination results of BI-RADS classification combined with shear wave elastography technology in routine ultrasound

#### 3.3.1 Shear wave elastography evaluation of breast nodules

The Young’s modulus values of breast nodules obtained through SWE measurement, including Emax, Emean, Emin, and SD, demonstrated substantial differences between the benign and malignant groups (P < 0.01) (**Table 2**).

**Table 2:** Comparison of SWE elasticity modulus parameters between benign and malignant breast lesions.

Pathology Result	Number	Emax/kPa	Emean/kPa	Emin/kPa	SD/kPa
Benign	50	42.95 ± 37.34	26.60 ± 21.95	15.69 ± 11.85	4.80 ± 6.04
Malignant	60	114.28 ± 23.83	66.40 ± 19.38	28.31 ± 11.86	13.39 ± 9.86
t value		12.133	10.099	5.555	5.369
P value		<0.001	<0.001	<0.001	<0.001

#### 3.3.2 Diagnostic performance of combined use of BI-RADS and shear wave elastography in breast nodule evaluation

**Figure 1:**
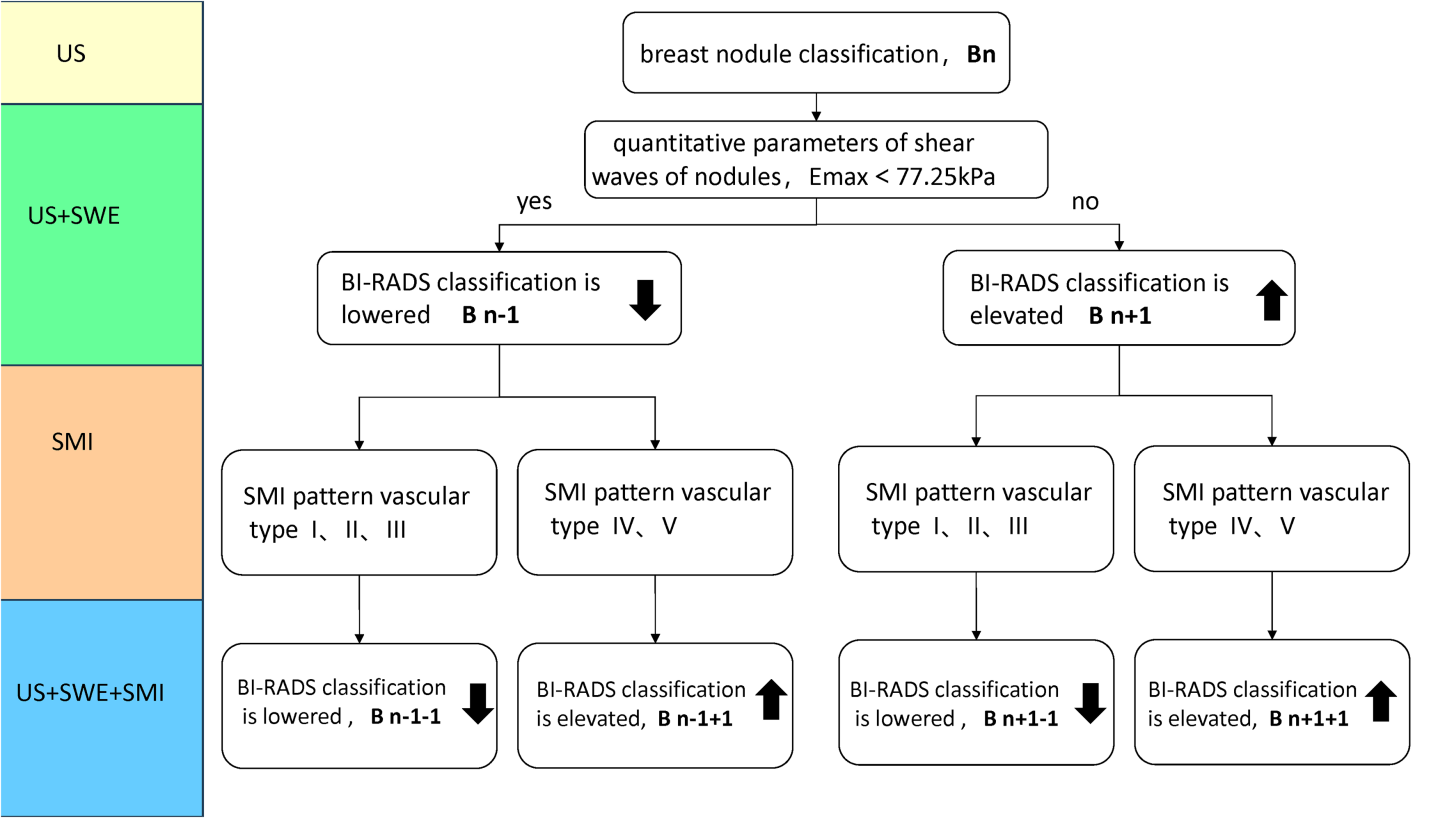
Classification Rule (Bn) for incorporating traditional ultrasound BI-RADS with SWE or SMI in **Figure 1**. When BI-RADS 3, Bn classification, n=1; BI-RADS-US 4a, Bn classification, n=2; BI-RADS 4b, Bn classification, n=3; BI-RADS 4c, Bn classification, n=4; BI-RADS 5, Bn classification, n=5. For instance, a nodule was initially classified as BI-RADS 3. Its Bn classification is 1, when Emax > 77.25kPa, it is classified as B2 (n+1) by adding SWE to traditional ultrasound (US + SWE); when the SMI mode is type III branch, it is classified as B1 (n+1-1), by adding SMI on US + SWE (US + SWE + SMI), Bn classification ranges from 1 to 5 after elevated and lowered. (SMI pattern vascular type: type I avascular, type II linear, type III branching, type IV root-like, type V crab-li.

In this research, BI-RADS classification and SWE were combined, with Emax ≥77.25 kPa and BI-RADS classification greater than 4a employed as the malignant standard. ROC curves were constructed for Emax (SWE), BI-RADS classification (US), and the combined diagnostic approach (US+SWE) (**Table 3**, **Figure 1**). The sensitivity of US+SWE was not substnatially different from that of US or SWE alone, while its NPV was slightly higher than that of the two individual classifications. The specificity, accuracy, and PPV of US and SWE substantially increased with the combined US+SWE approach. A statistically significant difference was observed in the AUC between the US+SWE approach and the US alone (Z = 3.404, P = 0.0007).

**Table 3:** Diagnostic performance of various examination methods for differentiating benign and malignant breast nodules.

Examination Method	Examination Result	Pathological Result (Number of Nodules)		Sensitivity (%)	Specificity (%)	Accuracy (%)	Positive Predictive Value (%)	Negative Predictive Value (%)	AUC
		Benign	Malignant						
US	Benign	38	2	96.67	76.00	82.27	82.86	95.00	0.938
	Malignant	12	58						
US+SWE	Benign	46	2	96.67	84.00	90.91	87.88	95.45	0.975
	Malignant	4	58						
US+SMI	Benign	46	3	95.00	92.00	93.63	93.44	93.88	0.966
	Malignant	4	57						
US+SWE+SMI	Benign	46	1	98.33	92.00	95.45	97.87	96.72	0.980
	Malignant	4	59						

### 3.4 Results of the examination using traditional ultrasound BI-RADS classification combined with super microvascular imaging technology

#### 3.4.1 Alder grading of breast nodule microvessels in SMI mode

In SMI mode, the blood flow classification of benign nodules based on the Alder grading system (**Table 4**) demonstrated statistically significant differences (χ^2 = ^44.153, P<0.01) compared with the pathological results. The microvascular morphology of benign nodules tended to be avascular [10 (19.4%)], linear [19 (38.00%)], or dendritic [19 (38.00%)], whereas malignant nodules tended to be root-like [20 (33.33%)] or crab-like [25 (41.67%)]. In SMI mode, there was a significant difference in microvascular morphology between benign and malignant breast nodules (χ2 = 56.181, P<0.01) (**Table 4**).

**Table 4:** Microvascular morphology classification of breast nodules under SMI mode [n (%)].

Pathological Type	Number	avascular	linear	dendritic	root-like	crab-like
Benign	50	10 (20)	19 (38)	19 (38)	1 (2)	1 (2)
Malignant	60	3 (5)	3 (5)	9 (15)	20 (33.33)	25 (41.67)
Total	110	13	22	28	21	26

#### 3.4.2 Optimization and adjustment of BI-RADS classification using SMI technology

In SMI mode, the BI-RADS classification remained unchanged or was downgraded when the microvascular morphology of breast nodules was classified as benign (avascular, linear, or dendritic). The BI-RADS classification remained unchanged or was upgraded when the microvascular morphology was classified as malignant (root-like or crab-like). The nodule classification remained unchanged when the low-level grayscale ultrasound showed a low-level microvascular classification and the high-level grayscale ultrasound showed a high-level microvascular classification. The classification was upgraded by one level when the low-level grayscale ultrasound showed a high-level microvascular classification. However, the classification could be downgraded by at most one level when the high-level grayscale ultrasound showed a low-level microvascular classification. **Table 5** shows a comparison of the adjusted BI-RADS classification with the pathological results.

**Table 5:** Comparison analysis of BI-RADS classification combined with SMI adjustment and pathological results for breast nodules (n).

BI-RADS Category	Nodules(n)		Pathological Result Benign		Pathological Result Malignant		Diagnostic Accuracy of Malignant Nodules (%)	
	Before Adjust	After Adjust	Before Adjust	After Adjust	Before Adjust	After Adjust	Before Adjust	After Adjust
3	26	37	26	37	0	0	0.00	0.00
4a	14	12	12	9	2	3	14.29	25.00
4b	27	19	10	2	17	17	62.96	89.47
4c	32	27	2	2	30	25	93.75	92.59
5	11	15	0	0	11	15	100.00	100.00
Total	110	110	50	50	60	60		

#### 3.4.3 Diagnostic performance of the adjusted BI-RADS classification for benign and malignant breast nodules

Compared with the traditional ultrasound BI-RADS classification (US) after adjustment, the adjusted BI-RADS classification employing SMI microvascular morphology classification (US+SMI) demonstrated a slightly lower sensitivity and NPV (P<0.05) (**Table 3**, **Figure 1**). However, compared with the prior adjustment, the specificity, accuracy, and PPV were significantly improved (χ2 = 4.763, P<0.05). The AUC for US+SMI was 0.966, and there was a statistically significant difference in the AUC between US+SMI and US (Z = 2.826, P = 0.0047). Combining SMI classification can enhance the diagnostic accuracy of benign and malignant breast nodules classified as BI-RADS 3-5.

### 3.5 The examination results of the traditional ultrasound BI-RADS classification combined with the SWE and SMI are concluded based on the following criteria

The BI-RADS classification is elevated or unchanged when Emax is greater than or equal to 77.25 kPa and/or the mSMI pattern is malignant vascular type (IV-type root-like or V-type crab-like). The BI-RADS classification is lowered or unchanged when Emax is less than 77.25 kPa and/or the SMI pattern is benign vascular type (I type avascular, II type linear, or III type branching) (**Figure 1**).

**Figure 2:**
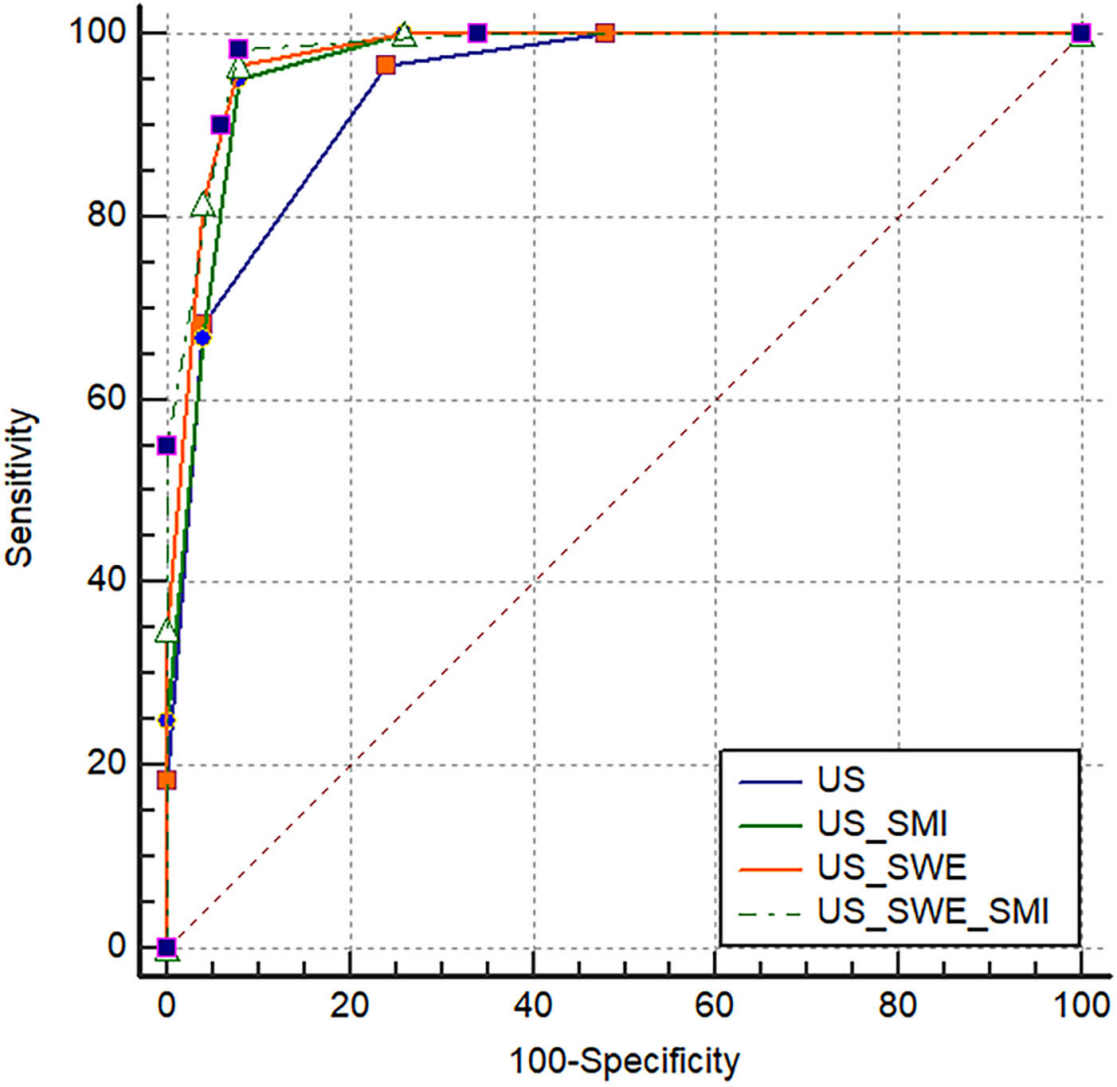
Receiver operating characteristic curves for US and three sets of combined tests.

Results showed that the AUC values of US+SWE+SMI, US+SWE, and US+SMI were 0.980, 0.975, and 0.966, respectively. Among them, the US+SWE+SMI had the highest AUC and diagnostic values (**Table 3**, **Figure 2**) compared to that of US-BI-RADS classification (P < 0.01). No statistical difference was observed in the AUC value between US+SWE+SMI and US+SWE or US+SMI. For distinguishing between benign and malignant breast nodules, the sensitivity, specificity, accuracy, PPV, and NPV of US+SWE+SMI were all superior to those of US, US+SWE, or US+SMI.

## 4 Discussion

**Figure 3:**
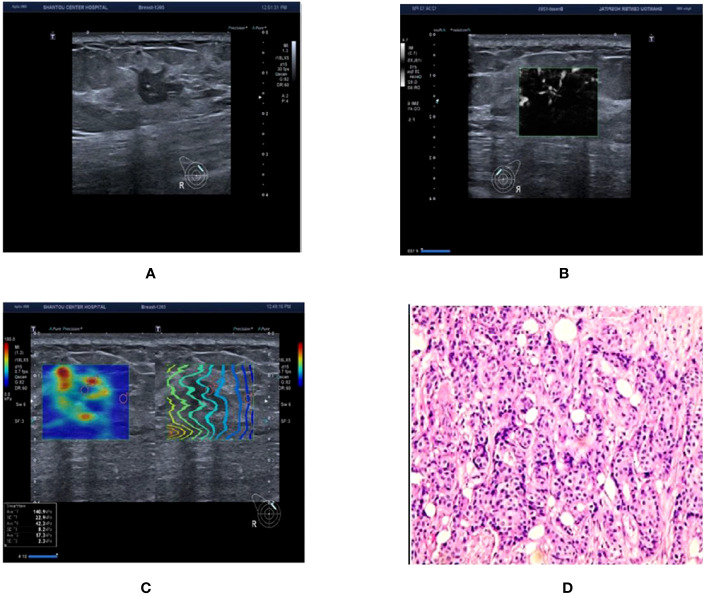
Ultrasound and pathological graphs: A 50-year-old female patient with a right breast nodule received ultrasound and pathological biopsy examinations. **(A)** An irregularly shaped, crab-like hypoechoic nodule is detected in the upper outer quadrant of the right breast, with unclear boundaries, classified as BI-RADS 4c. **(B)** mSMI image demonstrates that the microvascular morphology is radicle-shaped, with an increased BI-RADS level of 1 classified as BI-RADS 5. **(C)** Shear wave elastography image: Emax is 140.9 kPa, which is higher than 77.25 kPa, resulting in an increase or no change in the BI-RADS classification. **(D)** The nodule is an invasive ductal carcinoma based on pathological results.

**Figure 4:**
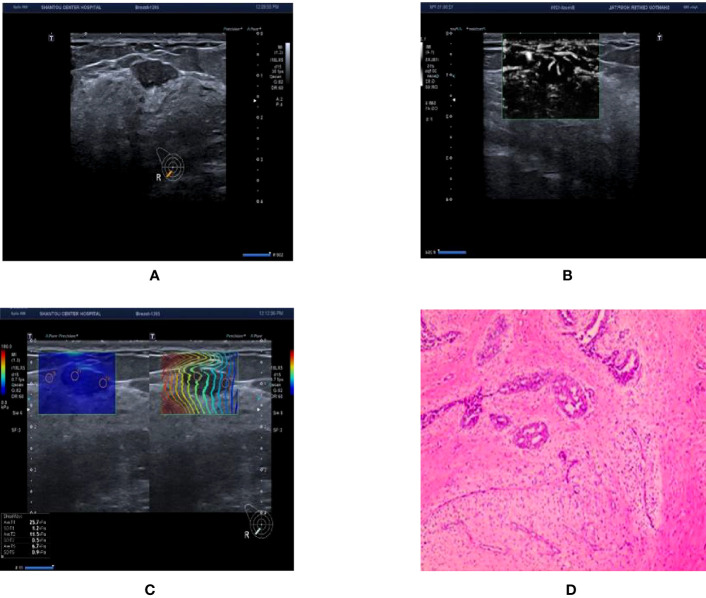
Ultrasound and pathological graphs. A 28-year-old female patient with a right breast nodule received ultrasound and pathological biopsy examinations. **(A)** A regular low-echo nodule was detected in the right outer and lower mammary quadrant, with parallel growth, lobed edges, even internal echo, and clear boundaries, BI-RADS 4a. **(B)** mSMI shows that microvessel morphology is dendritic subtype; BI-RADS grade was downgraded 1 level. **(C)** Shear wave elastic imaging: Emax is 25.7kPa < 77.25 kPa; BI-RADS classification was lowered or remained unaltered. **(D)** The nodule is a breast fibroadenoma based on pathological results.

To re-adjust BI-RADS classification based on the nodule hardness and blood flow characteristics of breast nodules, this research combined SWE and SMI technologies and further examined the diagnostic value of the two new technologies for discriminating benign and malignant BI-RADS 3-5 breast nodules (exmple **Figures 3**, **4**). This research discovered that the sensitivity, specificity, and accuracy of the BI-RADS classification adjusted by SMI and SWE technologies for the diagnosis of breast nodules were higher than those before adjustment (P<0.05). Combining SWE and SMI with traditional ultrasound BI-RADS classification can improve the diagnostic efficiency of BI-RADS 3-5 breast nodules, and their combination has the highest diagnostic efficiency, offering a more reliable diagnostic basis for clinical practice.

### 4.1 Classification ultrasound BI-RADS results

BI-RADS 3-4a nodules were classified as benign lesions and 4b-5 nodules were classified as malignant lesions based on the conventional ultrasound BI-RADS classification criteria in this experiment. The sensitivity of the traditional ultrasound BI-RADS classification for the diagnosis of benign and malignant breast nodules was high, but the specificity was low, indicating a high misdiagnosis rate of traditional ultrasound BI-RADS classification. Due to that, a large proportion of benign breast nodules will be misdiagnosed as malignant one. Some of the misdiagnosed benign lesions were fibroadenomas with active proliferation of surrounding ductal epithelium, and some fibroadenomas were accompanied by surrounding glandular disease, resulting in the misdiagnosis as malignant. Misdiagnosed malignant nodules had parallel growth, uniform internal echogenicity, slightly blurred margins, apparent capsules, and slight attenuation of posterior echoes; therefore, they were categorized as BI-RADS 4a. Particularly in BI-RADS 4, the malignancy rate of breast nodules previously examined using ultrasound as BI-RADS 3-5 varies significantly, and there were several similar morphological signs between benign and malignant nodules. Although diagnosed strictly according to the BI-RADS classification criteria, it is still challenging to accurately determine their classification sometimes. Furthermore, many patients with benign breast nodules have undergone excessive biological examination and surgery due to the misdiagnosis, which is unnecessary (27). Thus, it is urgent to develop diagnostic methods with high accuracy to reduce unnecessary clinical intervention.

### 4.2 Diagnostic performance of BI-RADS classification adjusted with SWE

This research combined SWE technology and BI-RADS classification criteria to exclude false-positive diagnoses of eight lesions in the BI-RADS classification standard. In cases where the lesion was small and the degree of fibrosis in the breast cancer cell matrix was low, the collagen fiber content decreased, resulting in a corresponding decrease in the hardness (28, 29). It has been reported that the higher breast thickness and lesion depth can result in lower elasticity values compared with actual values during SWE examination (19). Our results revleaed that the combination of US and SWE had an improved specificity and comparable sensitivity compared to US or SWQ alone. Hence, the addition of nodule elasticity contributes to the high diagnostic accuracy.

### 4.3 Diagnostic performance of BI-RADS classification adjusted with SMI

The blood flow levels of benign tumors were mostly at levels 0–1, while those of malignant tumors were mostly at levels 2–3. Malignant tumors have an abundant blood supply and a higher blood flow rate than benign tumors. Park et al. discovered that SMI was superior to color Doppler when evaluating the morphology, quantity and distribution of tumor microvessels (30). However, some fibroadenomas with large volume or quick growth rates will have similar vascular blood flow signals and penetrating vessel patterns compared to malignant tumors when using semiquantitative grading and penetrating vessels as diagnostic criteria (31, 32). Therefore, employing blood flow distribution pattern analysis as a diagnostic criterion is superior to semiquantitative grading in diagnosing benign and malignant breast lesions (33).

In this research, Adler grade 0-1 nodules with avascular, linear, dendritic subtype of microvascular morphology were classified as benign ones, while Adler grade 2-3 nodules with root and crab leg subtype of microvascular morphology were classified as malignant ones (34, 35). A significant difference of microvascular morphology classification and the Adler grade was observed between the benign and malignant breast nodules (P < 0.01). Furthermore, when we adjusted the BI-RADS classification in combination with SMI technology, four benign nodules were classified as malignant, two of which were classified as 4b type, both of which were sclerosing adenosis with poor blood supply. The other two cases were classified as 4c nodules, which were lymphocytic mastitis and chronic granulomatous mastitis, respectively, and both of them exhibited obvious malignant signs on traditional ultrasound with the Adler grade of 3 and the microvascular morphology of root and crab leg subtypes under the mSMI mode. Three malignant nodules were categorized as benign ones (BI-RADS 4a), of which two were verified to be ductal carcinoma in situ, and the other was verified to be infiltrating lobular carcinoma. Since traditional ultrasound only showed slight malignant signs, so downgrading blindly is not advisable. A total of 18 BI-RADS classifications of breast nodules were upgraded, and no downgrade was adopted. The combination of SMI and BI-RADS classification criteria can enhance the diagnostic accuracy of breast nodules but still has limitation.

In this research, 4b nodules showed avascular characteristics and were downgraded to 4a. Postoperative pathology demonstrated that they were breast glandular diseases with fibroadenoma. BI-RADS 4a nodules exhibited linear characteristics and were downgraded to category 3. Postoperative pathology indicated that they were fibroadenoma with peripheral proliferation. All two cases of 2D ultrasound images demonstrated irregular shapes and unclear borders. Among them, breast glandular disease with fibroadenoma was more common in clinical cases, with nodules frequently having unclear borders and a hard texture, which can be categorized as BI-RADS category 4. Six 4a nodules downgraded to grade 3 were all fibroadenomas, which were routinely classified as 4a because of their irregular morphology, some with angular or lobulated shapes but with a benign vascular pattern. According to the pathological findings, most of these patients were middle-aged to elderly women, and the nodules had been present in their bodies for numerous years without substantial changes in size. Pathological findings demonstrated that chronic inflammation around the edge of the nodules could result in irregular nodular morphology, and the blood flow distribution inside the nodules was not abundant. Therefore, the combination of SMI and traditional BI-RADS classification can help differentiate these modules.

### 4.4 Diagnostic performance of BI-RADS classification optimized by combining SWE and SMI technologies

In this research, 15 nodules were SWE-positive but SMI-negative, whereas 2 nodules were SWE-negative but SMI-positive. 48 nodules were positive for both technologies. When the findings were inconsistent, the BI-RADS category remained unchanged. The addition of SWE and SMI to the BI-RADS classification led to 26 fibroadenomas remaining unaltered, 9 fibroadenomas categorized as BI-RADS 4a being downgraded to level 3, and 8 benign 4b nodules being downgraded, indicating that not all level 4 nodules require immediate intervention or biopsy since they were benign nodules. However, five nodules were still misdiagnosed even after combining the SWE and SMI approaches.

## Data availability statement

The original contributions presented in the study are included in the article/supplementary material. Further inquiries can be directed to the corresponding author.

## Ethics statement

The studies involving humans were approved by Ethics Committee of Shantou Central Hospital. The studies were conducted in accordance with the local legislation and institutional requirements. Written informed consent for participation was not required from the participants or the participants’ legal guardians/next of kin because Approved by the Ethics Council.

## Author contributions

LW and XW designed the project. XW and YH drafted the manuscript. XW performed the experiments. XW and YH performed the data analysis. YH and LW critically reviewed and revised the manuscript. LW approved the submitted version of the manuscript. XW and YH contributed equally to this work.
